# Optimal Control for Aperiodic Dual-Rate Systems With Time-Varying Delays

**DOI:** 10.3390/s18051491

**Published:** 2018-05-09

**Authors:** Ernesto Aranda-Escolástico, Julián Salt, María Guinaldo, Jesús Chacón, Sebastián Dormido

**Affiliations:** 1 Departamento de Informática y Automática, Universidad Nacional de Educación a Distancia, 28040 Madrid, Spain; mguinaldo@dia.uned.es (M.G.); jchacon@bec.uned.es (J.C.); sdormido@dia.uned.es (S.D.); 2 Departamento de Ingeniería de Sistemas y Automática, Instituto Universitario de Automática e Informática Industrial, Universitat Politécnica de Valéncia, 46022 Valencia, Spain; julian@isa.upv.es

**Keywords:** multi-rate systems, optimization, aperiodic control, time-varying delay, air levitation system

## Abstract

In this work, we consider a dual-rate scenario with slow input and fast output. Our objective is the maximization of the decay rate of the system through the suitable choice of the *n*-input signals between two measures (periodic sampling) and their times of application. The optimization algorithm is extended for time-varying delays in order to make possible its implementation in networked control systems. We provide experimental results in an air levitation system to verify the validity of the algorithm in a real plant.

## 1. Introduction

This paper is defined in the context of multi-rate systems. Specifically, we consider a dual-rate system, where the output of the system is sampled with sampling period $T_{s}$ but the input is applied to the system in a faster way. Traditionally, if the controller transmits *n* input signals, these signals are applied during $T_{s}/n$ units of time (see [1,2] and references therein). In some cases, the times of application of the input signals might be irregular [3,4,5] but there are no criteria in the literature to decide this irregularity. In this work, we propose an optimization algorithm which provides not only the input signals but also their times of application, and maximizes the decay rate of the system.

Literature review: The theory of multi-rate control systems has been extensively studied [6,7,8] due to its multiple applications to real systems. For example, multi-rate control has been used for chemical analyzers [9,10] , visual feedback [11,12], flight control [13], or disk drive servo systems [14,15]. Its application in networked control systems is more recent [5,16,17] and has enabled a saving in communication resources since similar performance can be achieved with a reduced number of transmissions. From the point of view of the optimization problem, optimal controllers have been designed in the framework of multi-rate systems. In [18] an $H_{2}$ optimal control is described. A periodic optimal control is proposed in [19]. In [20], an optimal controller is designed based on linear matrix inequality optimization. Several predictive controllers have been studied in [21,22,23,24]. However, even when these works propose different optimal controllers, they do not consider irregular sampling or time-varying delays. In [25], the predictive control for multi-rate systems is extended to the distributed case. However, even when these works propose different optimal controllers, they consider neither irregular sampling nor time-varying delays. Recently, the ideas of minimum attention control [26,27] and anytime attention control [28,29] have been studied for networked control systems. In the minimum attention control, the time of application of the control signal is maximized while a certain level of performance is maintained. In anytime attention control, the time of application is previously fixed depending on the available resources and the performance is maximized for that period. The method proposed in this work attempts, in some way, to carry out a combination of both. In the algorithm we maximize the decay rate of the system after a full sampling period of the output, which is fixed, however within this interval of time we vary the times of application as desired to not only maintain the performance but also to improve it.

Statement of contributions: In the present work, we continue the ideas proposed in [30]. In [30], we developed a dual-rate controller to maximize the decay rate of the system through the computation of optimal input signals. In this work, we extend the framework in four directions. Firstly, we consider the optimization not only of the values of the input signals but also of their times of application. This implies obtaining an aperiodic controller that decides the times of application, which is significantly different with respect to other multi-rate aperiodic solutions (see [3] and references therein). Secondly, we study the optimization problem under a model plant mismatching scenario, which makes the algorithm more applicable to real processes. Thirdly, while in [30] constant delays are considered, in this work we extend the solutions for time-varying delays. This approach is more realistic from the point of view of networked control systems [31]. Fourthly, we provide guidelines to implement the optimization algorithm in real systems with limited computation resources and show satisfactory experimental results controlling an air levitation system. Therefore, the optimization with respect to the times of application considering time-varying delays offers not only a larger decay rate than in [30] but also allows dealing with the effect of the delays more efficiently. The contributions of the paper with respect to the literature can be seen from different perspectives. The proposed algorithm is the only one, to the best of our knowledge, that combines optimization and irregular sampling. Hence, the proposed algorithm implies more degrees of freedom in the optimization than other multi-rate optimization algorithms [19,20,21,22,23,24] because it considers the times of application. Moreover, it provides a criterion to efficiently decide the form of this irregular sampling, which differs from other irregular sampling schemes [3,4,5], where the times of application are predetermined.

Organization: The reminder of the paper is organized as follows. In Section 2, the problem is presented and the lifted multi-rate model is developed. In Section 3, the proposed algorithm is presented and we extend the results for time-varying delay systems. A discussion about how to perform the optimization more efficiently in systems with hard computation constraints is also provided. In Section 4, the experimental results are provided. Finally, a discussion about the benefits of the method is given in Section 5.

### Preliminaries

We define the set of real numbers and the set of natural numbers as R and N, respectively. The *n*-dimensional real space is defined by $R^{n}$. We refer to the euclidean norm of vector $x\in R^{n}$ as $∥x∥=\sqrt{x^{⊤}x}$. Let $M\in R^{n\times m}$; $M^{⊤}$ denotes the transpose matrix of *M*. In addition, if *M* is a symmetric real matrix, then the maximum and the minimum eigenvalue of *M* are denoted by $\lambda_{\max}\left(M\right)$ and $\lambda_{\min}\left(M\right)$, respectively. We further denote a symmetric positive-definite matrix $P\in R^{n\times n}$ as P>0, while P≥0, P<0, and P≤0 refer to symmetric positive-semidefinite, negative-definite, and negative-semidefinite matrices, respectively. We denote the identity matrix $I\in R^{n\times n}$ by $I_{n}$. Let $A\in R^{n\times n}$ and $B\in R^{n\times m}$. We define $\mu \left(A\right)=\max\left\{\mu |\mu \in \lambda \left((A+A^{⊤})/2\right)\right\}$. The norm of the matrix exponential [32] can be bounded then such that

(1) $$∥e^{A\theta }∥\leq e^{\mu (A)\theta }\leq e^{\mu (A)(\theta +\epsilon )},\;\forall \epsilon \geq 0.$$

B(θ,A) denotes

(2) $$B\left(\theta ,A\right)=\left\{\begin{array}{cc} 0 & \mathrm{if}\;\theta \leq 0\\ \int_{0}^{\theta }e^{As}Bds & \mathrm{if}\;\theta >0 \end{array}\right.$$

and $B_{\mu }\left(\theta ,A\right)$

$$B_{\mu }\left(\theta ,A\right)=\left\{\begin{array}{cc} 0 & \mathrm{if}\;\theta \leq 0\\ \int_{0}^{\theta }e^{\mu (A)s}∥B∥ds & \mathrm{if}\;\theta >0 \end{array}\right..$$

Consequently,

(3) $$∥B\left(\theta ,A\right)∥\leq B_{\mu }\left(\theta ,A\right)\leq B_{\mu }\left(\theta +\epsilon ,A\right),\;\forall \epsilon \geq 0.$$

Finally, we define the exponential stability of a system

(4) $$\dot{x}\left(t\right)=f\left(t,x\right)$$

where $f:\left[0,\infty \right)\times D\to R^{n}$ is piecewise continuous in *t* and locally Lipschitz in *x* on [0,∞)×D, and $D\in R^{n}$ is a domain that contains the origin x=0 as follows [33]:

**Definition** **1.** *The equilibrium point x=0 of *(4)* is exponentially stable if there exist positive constants ϵ, c, and α such that*

(5) $$∥x\left(t\right)∥\leq ce^{-\alpha \left(t-t_{0}\right)}∥x\left(t_{0}\right)∥,\;\forall ∥x\left(t_{0}\right)∥<\epsilon$$

*and globally exponentially stable if *(5)* is satisfied for any initial state $x(t_{0})$.*

## 2. Problem Statement

**Figure 1:**
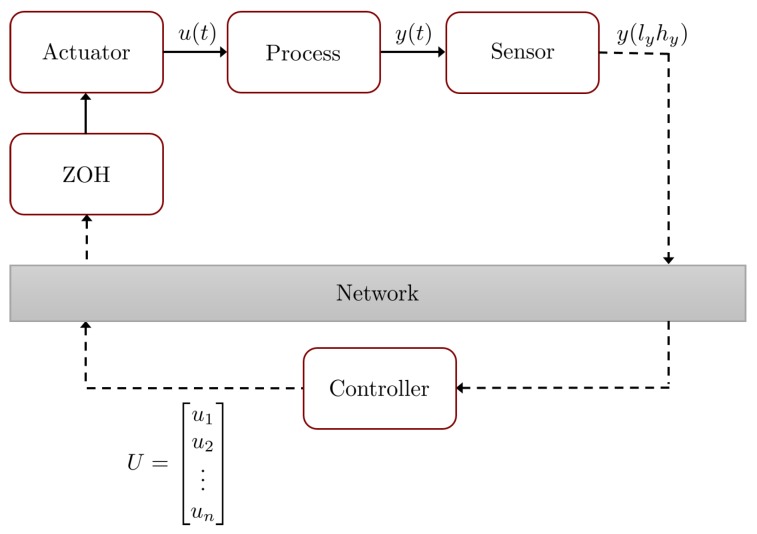
Block diagram of a dual-rate system with fast input.

Let us consider a continuous linear time-invariant (LTI) plant denoted by

(6) $$\begin{array}{c} {\dot{x}}_{p}\left(t\right)=A_{p}x_{p}\left(t\right)+B_{p}u\left(t\right)\\ y\left(t\right)=C_{p}x_{p}\left(t\right) \end{array}$$

with $x_{p}\left(t\right)\in R^{n_{x_{p}}}$ the state vector of the plant, $u\left(t\right)\in R^{n_{u}}$ the input vector, $y\left(t\right)\in R^{n_{y}}$ the output vector, and $A_{p}\in R^{n_{x_{p}}\times n_{x_{p}}}$, $B_{p}\in R^{n_{x_{p}}\times n_{u}}$, and $C_{p}\in R^{n_{y}\times n_{x_{p}}}$ the constant matrices. We assume that the output of (6) is sampled with sampling period $T_{s}$ as shown in Figure 1. In contrast, the controller changes the input signal *n* times during $T_{s}$. Let us assume that each input $u_{i}$ for i=1,...,n remains constant using a zero-order-hold (ZOH) and is applied during the period of time $T_{i}$ satisfying $T_{s}=\sum_{i=1}^{n}T_{i}$. Then,

(7) $$u\left(t\right)=\left\{\begin{array}{c} u_{1}\;\mathrm{if}\;t\in \left[kT_{s},kT_{s}+T_{1}\right)\\ u_{2}\;\mathrm{if}\;t\in \left[kT_{s}+T_{1},kT_{s}+T_{1}+T_{2}\right)\\ \vdots \\ u_{n}\;\mathrm{if}\;t\in \left[kT_{s}+\sum_{i=1}^{n-1}T_{i},kT_{s}+\sum_{i=1}^{n}T_{i}\right) \end{array}\right..$$

The discretization of (6)–(7) leads to a periodic linear time-varying discrete system, which implies different problems in order to guarantee the stability and, consequently, in order to maximize the decay rate. A way to avoid this is through lifting techniques [7,8]. We can write the dual-rate sampling scenario in a lifted fashion as

(8) $$x_{p}\left(t\right)=e^{A_{p}\left(t-T_{s}\right)}x_{p}\left(k\right)+\sum_{i=1}^{n}B_{p}^{i}\left(t-T_{s},A_{p}\right)u_{i}$$

with

(9) $$\begin{array}{c} B_{p}^{i}\left(t-T_{s},A_{p}\right)=e^{A_{p}\left(t-T_{s}-\min\left(t-T_{s},\sum_{j=0}^{i}T_{j}\right)\right)}\times B_{p}\left(\min\left(t-T_{s}-\sum_{j=0}^{i-1}T_{j},T_{i}\right),A_{p}\right). \end{array}$$

Consequently, the state after a whole sampling period of the output is

(10) $$x_{p}\left(\left(k+1\right)T_{s}\right)={\tilde{A}}_{p}x_{p}\left(kT_{s}\right)+{\tilde{B}}_{p}U$$

with $U={\left[\begin{array}{ccc} u_{1} & \cdots & u_{n} \end{array}\right]}^{⊤}$, ${\tilde{A}}_{p}=e^{A_{p}T_{s}}$, and ${\tilde{B}}_{p}=\left[\begin{array}{ccc} B_{p}^{1}\left(T_{s},A_{p}\right) & \cdots & B_{p}^{n}\left(T_{s},A_{p}\right) \end{array}\right]$.

## 3. Control Algorithm for Decay Rate Optimization

In this section, we design the algorithm to maximize the decay rate of the system. A priori, the decay rate of a system can be maximized as much as desired if the input signal is sufficiently large. Naturally, this is not an appropriate solution for a real system, where the actuator might saturate the input signal or might not allow fast changes. To overcome this issue, we consider an auxiliary LTI controller for which we guarantee global asymptotic stability with a given maximum gain. Then, we provide the necessary constraints to the optimization problem so that the decay rate is maximized maintaining this maximum gain.

For the optimization of the decay rate, it is necessary to ensure the exponential stability of the system. With this motivation, let us recall the following Lemma.

**Lemma** **1.** *The exponential stability of the discretized plant *(10)* is guaranteed with decay rate $\hat{\alpha }>0$, if there exists a Lyapunov function $V\left(x_{p}\left(kT_{s}\right)\right)=V\left(k\right):R^{n_{x_{p}}}\to R$ and positive scalars $\lambda_{1}$, $\lambda_{2}$ and positive integer q such that*

(11) $$\lambda_{1}∥x_{p}{\left(k\right)∥}^{q}\leq V\left(k\right)\leq \lambda_{2}{∥x_{p}\left(k\right)∥}^{q}$$

*and*

(12) $$V\left(k+1\right)-e^{q\hat{\alpha }T_{s}}V\left(k\right)\leq 0.$$

Next, to compute the input signals $\left\{u_{i}\right\}$ and their times of application $\left\{T_{i}\right\}$, we require an estimation of the model of the plant in order to obtain an estimated set of outputs $\left\{y_{pm,i}\right\}$. Let us consider that the model of the plant is described by the estimated state $x_{pm}$, and let us define the matrices $A_{pm},B_{pm},C_{pm}$ analogously to (6). Then, this produces the output

$$\begin{array}{cc} y_{pm,1} & =y_{p}\left(kT_{s}\right)\;\mathrm{for}\;t\in \left[kT_{s},\left(k+1\right)T_{s}\right)\\ y_{pm,i} & =C_{pm}x_{pm}\left(kT_{s}+\sum_{j=0}^{i-1}T_{j}\right)\;\mathrm{for}\;t\in \left[kT_{s},\left(k+1\right)T_{s}\right)\;\mathrm{and}\;1<i\leq n. \end{array}$$

Let us consider that there exists an auxiliary LTI controller as

(13) $$\begin{array}{cc} {\dot{x}}_{c}\left(t\right) & =A_{c}x_{c}\left(t\right)+B_{c}e\left(t\right)\\ \hat{u}\left(t\right) & =C_{c}x_{c}\left(t\right)+D_{c}e\left(t\right), \end{array}$$

where e(t)=r(t)−y(t), with r(t) being the reference signal (we can take r(t)=0 for simplicity), $x_{c}\left(t\right)\in R^{n_{x_{c}}}$ the state vector of the controller, $\hat{u}\left(t\right)\in R^{n_{u}}$ the auxiliary input vector, and $A_{c}\in R^{n_{x_{c}}\times n_{x_{c}}}$, $B_{c}\in R^{n_{x_{c}}\times n_{y}}$, $C_{c}\in R^{n_{u}\times n_{x_{c}}}$, and $D_{c}\in R^{n_{u}\times n_{y}}$ the constant matrices. In a multi-rate scenario and using a lifted framework, a virtual closed loop of the augmented state ${\hat{x}}^{⊤}={\left[\begin{array}{cc} x_{p} & x_{c} \end{array}\right]}^{⊤}$ can be written as

(14) $$\hat{x}\left(\left(k+1\right)T_{s}\right)=\Pi \left({\hat{T}}_{1},...,{\hat{T}}_{n}\right)\hat{x}\left(kT_{s}\right),$$

where ${\hat{T}}_{i}$ is the time of application of ${\hat{u}}_{i}$, and

(15) $$\begin{array}{c} \Pi ({\hat{T}}_{1},...,{\hat{T}}_{n})=e^{AT_{s}}+e^{A\left(T_{s}-{\hat{T}}_{1}\right)}B\left({\hat{T}}_{1},A\right)K\\ +\sum_{i=2}^{n}e^{A\left(T_{s}-\sum_{j=1}^{i}{\hat{T}}_{j}\right)}B\left({\hat{T}}_{i},A\right)K_{m}\prod_{j=1}^{i}\left(e^{A_{m}{\hat{T}}_{i-j}}+B_{m}\left({\hat{T}}_{i-j},A_{m}\right)K_{m}\right). \end{array}$$

$A=\left[\begin{array}{cc} A_{p} & 0\\ 0 & A_{c} \end{array}\right]$, $B=\left[\begin{array}{cc} B_{p} & 0\\ 0 & B_{c} \end{array}\right]$, $K=\left[\begin{array}{cc} -D_{c}C_{p} & C_{c}\\ -C_{p} & 0 \end{array}\right]$, $A_{m}=\left[\begin{array}{cc} A_{pm} & 0\\ 0 & A_{c} \end{array}\right]$, $B_{m}=\left[\begin{array}{cc} B_{pm} & 0\\ 0 & B_{c} \end{array}\right]$, $K_{m}=\left[\begin{array}{cc} -D_{c}C_{pm} & C_{c}\\ -C_{pm} & 0 \end{array}\right]$ and ${\hat{T}}_{0}=T_{0}=0$.

We further introduce the following assumption:

**Assumption** **1.** *Matrices $C_{c}$ and $D_{c}$, a positive definite matrix P, and scalars ${\hat{T}}_{1},...,{\hat{T}}_{n}\geq 0$ exist such that*

(16) $$\sum_{i=1}^{n}{\hat{T}}_{n}=T_{s}$$

(17) $$\Pi^{⊤}\left({\hat{T}}_{1},...,{\hat{T}}_{n}\right)P\Pi \left({\hat{T}}_{1},...,{\hat{T}}_{n}\right)-e^{-2\hat{\alpha }T_{s}}P\leq 0,$$

*are satisfied for some $\hat{\alpha }>0$.*

Then, the following theorem can be stated.

**Theorem** **1.** *Consider the auxiliary discretized closed loop system *(14)*. Suppose that Assumption 1 holds. Then, *(14)* is globally exponentially stable with, at a minimum, a decay rate of $\hat{\alpha }>0$ and a gain*

(18) $$\begin{array}{c} c\left({\hat{T}}_{1},...,{\hat{T}}_{n}\right)=\sqrt{\frac{\lambda_{\max}\left(P\right)}{\lambda_{\min}\left(P\right)}}e^{\hat{\alpha }T_{s}}(e^{\mu \left(A\right)T_{s}}+e^{\mu \left(A\right)\left(T_{s}-{\hat{T}}_{1}\right)}B_{\mu }\left({\hat{T}}_{1},A\right)∥K∥\\ +\sum_{i=2}^{n}e^{\mu \left(A\right)\left(T_{s}-\sum_{j=1}^{i}{\hat{T}}_{j}\right)}B_{\mu }\left({\hat{T}}_{i},A_{p}\right)∥K_{m}∥\prod_{j=1}^{i}\left(e^{\mu \left(A_{m}\right){\hat{T}}_{i-j}}+{B_{m}}_{\mu }\left({\hat{T}}_{i-j},A_{m}\right)∥K_{m}∥\right)). \end{array}$$

**Proof** **of** **Theorem** **1.** Consider a Lyapunov function of the form

(19) $$V\left(kT_{s}\right)={\hat{x}}^{⊤}\left(kT_{s}\right)P\hat{x}\left(kT_{s}\right)$$

where P>0. Then,

$$\lambda_{\min}\left(P\right)∥\hat{x}\left(kT_{s}\right){∥}^{2}\leq V\left(kT_{s}\right)\leq \lambda_{\max}\left(P\right){∥\hat{x}\left(kT_{s}\right)∥}^{2}$$

is satisfied and (11) is fulfilled. The exponential decrease of the system (14) with decay rate $\hat{\alpha }$ for some ${\hat{T}}_{1},...,{\hat{T}}_{n}$ is achieved if (12) in Lemma 1 is satisfied, i.e., if

(20) $$\begin{array}{c} V\left(\left(k+1\right)T_{s}\right)-e^{-2\hat{\alpha }T_{s}}V\left(kT_{s}\right)={\hat{x}}^{⊤}\left(kT_{s}\right)\left(\Pi^{⊤}\left({\hat{T}}_{1},...,{\hat{T}}_{n}\right)P\Pi \left({\hat{T}}_{1},...,{\hat{T}}_{n}\right)-e^{-2\hat{\alpha }T_{s}}P\right)\hat{x}\left(kT_{s}\right)\leq 0, \end{array}$$

which is guaranteed by (17). To find $c({\hat{T}}_{1},...,{\hat{T}}_{n})$, we use (19) and (20). From (20),

$$V\left(\left(k+1\right)T_{s}\right)\leq e^{-2\hat{\alpha }T_{s}}V\left(kT_{s}\right)\leq \cdots \leq e^{-2\hat{\alpha }kT_{s}}V\left(0\right)$$

and using (19), we obtain

$$∥\hat{x}\left(k\right)∥\leq \sqrt{\frac{\lambda_{\max}\left(P\right)}{\lambda_{\min}\left(P\right)}}e^{-\hat{\alpha }kT_{s}}∥\hat{x}\left(0\right)∥.$$

The state of the plant in any instance of time $t=kT_{s}+\delta$, $\forall \delta \in [0,T_{s}]$ is also exponentially stable since it can be bounded in the view of (8) as follows

(21) $$\begin{array}{cc} ∥\hat{x}\left(t\right)∥ & \leq (∥e^{AT_{s}}∥+∥e^{A\left(T_{s}-{\hat{T}}_{1}\right)}∥B\left({\hat{T}}_{1},A\right)∥∥K∥\\ & +\sum_{i=2}^{n}e^{∥A∥\left(T_{s}-\sum_{j=1}^{i}{\hat{T}}_{j}\right)}∥B\left({\hat{T}}_{i},A\right)∥∥K_{m}∥\prod_{j=1}^{i}\left(∥e^{A_{m}T_{i-j}}∥+∥B_{m}\left({\hat{T}}_{i-j},A_{m}\right)∥∥K_{m}∥\right))∥\hat{x}\left(kT_{s}\right)∥ \end{array}$$

Since $∥\hat{x}\left(kT_{s}+\sum_{j=0}^{i}T_{j}\right)∥$ can be bounded recursively until obtaining a bound which depends on $\hat{x}\left(0\right)$, and using (1) and (3), we can write

(22) $$\begin{array}{cc} ∥\hat{x}\left(t\right)∥ & \leq \tilde{c}\left({\hat{T}}_{1},...,{\hat{T}}_{n}\right)∥\hat{x}\left(kT_{s}\right)∥\leq \tilde{c}\left({\hat{T}}_{1},...,{\hat{T}}_{n}\right)\sqrt{\frac{\lambda_{\max}\left(P\right)}{\lambda_{\min}\left(P\right)}}e^{-\hat{\alpha }kT_{s}}∥\hat{x}\left(0\right)∥\\ & =c\left({\hat{T}}_{1},...,{\hat{T}}_{n}\right)e^{-\hat{\alpha }\left(t\right)}∥\hat{x}\left(0\right)∥. \end{array}$$

Thus, the proof is completed.  ☐

Having proved that there exists a controller able to globally exponentially stabilize (14), we can design the input signals and their times of application, which maximize the decay rate of the system (10). As aforementioned, maximizing the decay rate enlarges the gain. Therefore, we need to establish constraints to maintain *c* in acceptable values. Let us consider the following assumptions in order to guarantee the optimization of the decay rate while satisfying these conditions.

**Assumption** **2.** *A bound $\bar{c}$ can be computed such that $\bar{c}=\max\left(c\left({\hat{T}}_{1},...,{\hat{T}}_{n}\right)\right)$.*

**Assumption** **3.** *The set of control signals $\left\{u_{i}\right\}$ for i=1,...,n satisfies that*

(23) $$\begin{array}{cc} ∥u_{1}∥ & \leq ∥K∥∥x\left(kT_{s}\right)∥ \end{array}$$

(24) $$\begin{array}{cc} ∥u_{i}∥ & \leq ∥K_{m}∥∥x_{m}\left(kT_{s}+\sum_{j=0}^{i-1}T_{j}\right)∥,\;for\;i>1. \end{array}$$

Then, the following theorem can be stated.

**Theorem** **2.** *Consider the discretized closed loop system *(10)* and control signals $\left\{u_{i}\right\}$ applied during periods $\left\{T_{i}\right\}$ for i=1,...,n. Suppose that Assumption 2 and 3 hold. Then, the system *(10)* is globally exponentially stable with at least decay rate $\alpha \geq \hat{\alpha }$ and gain $\bar{c}$.*

**Proof** **of** **Theorem** **2.** By Assumption 2, $\bar{c}\geq c\left(T_{1},...,T_{n}\right)$. From Theorem 1 it is guaranteed at least $\alpha =\hat{\alpha }$ if $u_{i}={\hat{u}}_{i}$ and $T_{i}={\hat{T}}_{i}$ for i=1,...,n. Equations (23) and (24) from Assumption 3 imply that ∥x(t)∥, for $t\in [kT_{s},\left(k+1\right)T_{s})$, satisfies (in the view of (21))

(25) $$\begin{array}{cc} ∥x(t)∥ & \leq (∥e^{AT_{s}}∥+∥e^{A\left(T_{s}-T_{1}\right)}∥B\left(T_{1},A\right)∥∥K∥\\ & +\sum_{i=1}^{n}e^{∥A∥\left(T_{s}-\sum_{j=2}^{i}T_{j}\right)}∥B\left(T_{i},A\right)∥∥K_{m}∥\\ & \times \prod_{j=1}^{i}\left(∥e^{A_{m}T_{i-j}}∥+∥B_{m}\left(T_{i-j},A_{m}\right)∥∥K_{m}∥\right))∥x\left(kT_{s}\right)∥\\ & \leq c\left(T_{1},...,T_{n}\right)∥x\left(0\right)∥\leq \bar{c}∥x\left(0\right)∥. \end{array}$$

Hence, Definition 1 is fulfilled for the closed loop system. If we consider that the initial state of the auxiliary controller is zero, then

(26) $$∥x_{p}\left(t\right)∥\leq \bar{c}e^{\alpha (t)}∥x_{p}\left(0\right)∥,$$

Thus, the process is globally exponentially stable with, at least, decay rate α and gain $\bar{c}$ and the proof is completed.  ☐

**Remark** **1.** *If we assume that the model matches with the actual system dynamics, i.e., if we assume that $A_{m}=A_{p}$, $B_{m}=B_{p}$ and $C_{m}=C_{p}$, then $\bar{c}=c\left(T,...,T\right)$, where $T=T_{s}/n$ (see Appendix A).*

### 3.1. Extension for Time-Varying Delay Systems

In this section, the results from Section 3 are formalized for the case with time-varying delays. Delays might affect to a control system in different ways. In our case, there are two main sources of delays. On the one hand, the transmission of information through the network might cause time-varying delays. On the other hand, the computation of the solutions of the optimization problem also induces a delay $\tau_{com}$, which we can assume constant [34]. Let us denote the transmission delays from the sensor to the controller and from the controller to the sensor as $\tau_{sc}$, $\tau_{ca}$, respectively, and let us assume that they are upper bounded as $0\leq \tau_{sc}\leq \tau_{M,net}$ and $0\leq \tau_{ca}\leq \tau_{M,net}$. Hence, the input signals in a meta-period *k* reach the actuator with a total delay τ(k), which is bound such that $\tau_{com}\leq \tau \left(t\right)\leq \tau_{com}+2\tau_{M,net}=\tau_{M}$. To simplify the stability analysis, let us consider a minimum unit of delay $\tau_{m}$ such that $\tau_{M}=l\tau_{m}$ for l≥1. Taking this into account, we consider that the actuator waits to apply the inputs signals until the next multiple of $\tau_{m}$ is larger than τ(k), i.e., the actual delay applied to the plant is $\hat{\tau }\left(k\right)=\min\left\{l\tau_{m}|\tau \left(k\right)\leq l\tau_{m}\right\}$.

Now, we follow the description in [5,35] to deal with delays in multi-rate systems. Let us assume that during the delay, the last input signal of the previous output sampling period is held. Let us also consider a vector ψ(k) which corresponds to the last input signal in the previous metaperiod. Then, we can define the following augmented state

(27) $$\xi \left(t\right)=\left(\begin{array}{c} x(t)\\ \psi (k) \end{array}\right),\;\forall t\in \left[kT_{s},\left(k+1\right)T_{s}\right).$$

As in Section 3, the auxiliary controller (13) is used to define a virtual closed loop

(28) $$\begin{array}{cc} \hat{\xi }\left(\left(k+1\right)T_{s}\right) & =\Pi_{d}\hat{\xi }\left(kT_{s}\right)=\left[\begin{array}{cc} \Pi_{d}^{11}\left(T_{0},{\hat{T}}_{1},...,{\hat{T}}_{n}\right) & \Pi_{d}^{12}\left(T_{0},{\hat{T}}_{1},...,{\hat{T}}_{n}\right)\\ \Pi_{d}^{21}\left(T_{0},{\hat{T}}_{1},...,{\hat{T}}_{n}\right) & \Pi_{d}^{22}\left(T_{0},{\hat{T}}_{1},...,{\hat{T}}_{n}\right) \end{array}\right]\hat{\xi }\left(k\right) \end{array}$$

where ${\hat{\xi }}^{⊤}\left(k\right)=\left(\begin{array}{cc} \hat{x}\left(k\right) & \psi (k) \end{array}\right)$ and

$$\begin{array}{c} \Pi_{11}^{d}\left(T_{0},{\hat{T}}_{1},...,{\hat{T}}_{n}\right)=e^{AT_{s}}+\sum_{i=1}^{n}e^{A\left(T_{s}-\sum_{j=1}^{i}{\hat{T}}_{j}\right)}B\left({\hat{T}}_{i},A\right)K_{m}\times \prod_{j=1}^{i}\left(e^{A_{m}{\hat{T}}_{i-j}}+B_{m}\left({\hat{T}}_{i-j},A_{m}\right)K_{m}\right)e^{A_{m}T_{0}}\\ \Pi_{12}^{d}\left(T_{0},{\hat{T}}_{1},...,{\hat{T}}_{n}\right)=e^{A\left(T_{s}-T_{0}\right)}B\left(T_{0},A\right)+\sum_{i=1}^{n}e^{A\left(T_{s}-\sum_{j=1}^{i}{\hat{T}}_{j}\right)}B\left({\hat{T}}_{i},A\right)K_{m}\times \prod_{j=1}^{i}\left(e^{A_{m}{\hat{T}}_{i-j}}+B_{m}\left({\hat{T}}_{i-j},A_{m}\right)K_{m}\right)B_{m}\left(T_{0},A_{m}\right) \end{array}$$

$$\begin{array}{c} \Pi_{21}^{d}\left(T_{0},{\hat{T}}_{1},...,{\hat{T}}_{n}\right)=K_{m}\left(e^{AT_{s}}+\sum_{i=1}^{n-1}e^{A\left(T_{s}-\sum_{j=1}^{i}{\hat{T}}_{j}\right)}B\left({\hat{T}}_{i},A\right)K_{m}\right.\left.\times \prod_{j=1}^{i}\left(e^{A_{m}{\hat{T}}_{i-j}}+B_{m}\left({\hat{T}}_{i-j},A_{m}\right)K_{m}\right)e^{A_{m}T_{0}}\right)\\ \Pi_{22}^{d}\left(T_{0},{\hat{T}}_{1},...,{\hat{T}}_{n}\right)=K_{m}\left(e^{A\left(T_{s}-T_{0}\right)}B\left(T_{0},A\right)+\sum_{i=1}^{n-1}e^{A\left(T_{s}-\sum_{j=1}^{i}{\hat{T}}_{j}\right)}B\left({\hat{T}}_{i},A\right)K_{m}\right.\left.\times \prod_{j=1}^{i}\left(e^{A_{m}{\hat{T}}_{i-j}}+B_{m}\left({\hat{T}}_{i-j},A_{m}\right)K_{m}\right)B_{m}\left(T_{0},A_{m}\right)\right), \end{array}$$

where $T_{0}=\hat{\tau }\left(k\right)$. Then, we can use again Lemma 1 to guarantee the exponential stability of (28). Let us recall and extend Assumption 1 for the augmented state $\hat{\psi }\left(k\right)$.

**Assumption** **4.** *Matrices $C_{c}$ and $D_{c}$, a positive definite matrix P, and scalars $\tau_{m}$, ${\hat{T}}_{1,l}=\tau_{M}-l\tau_{m}$, ${\hat{T}}_{2},...,{\hat{T}}_{n}\geq 0$ exist such that*

(29) $$l\tau_{m}+{\hat{T}}_{1,l}+\sum_{i=2}^{n}{\hat{T}}_{n}=T_{s}$$

(30) $$\Pi_{d}^{⊤}\left(l\tau_{m},{\hat{T}}_{1,l},{\hat{T}}_{2}...,{\hat{T}}_{n}\right)P\Pi_{d}\left(l\tau_{m},{\hat{T}}_{1,l},{\hat{T}}_{2}...,{\hat{T}}_{n}\right)-e^{-2\hat{\alpha }T_{s}}P\leq 0,$$

*are satisfied for some $\hat{\alpha }>0$ and for all l∈N such that $\tau_{m}\leq l\tau_{m}\leq \tau_{M}$.*

The following Theorem is formulated.

**Theorem** **3.** *Consider the auxiliary discretized closed loop system *(28)*. Suppose that Assumption 1 holds. Then, *(28)* is globally exponentially stable with, at least, a decay rate $\hat{\alpha }>0$ and gain*

(31) $$\hat{c}=\sqrt{\frac{\lambda_{\max}\left(P\right)}{\lambda_{\min}\left(P\right)}}e^{\hat{\alpha }T_{s}}\left({\hat{c}}_{1}+{\hat{c}}_{2}\right),$$

*where ${\hat{c}}_{i}=\max\left(c_{i}\left(l\tau_{m},{\hat{T}}_{1,l},{\hat{T}}_{2},...,{\hat{T}}_{n}\right)\right)$ for i=1,2, and*

$$\begin{array}{cc} c_{1}\left(T_{0},{\hat{T}}_{1},...,{\hat{T}}_{n}\right) & =e^{AT_{s}}+\sum_{i=1}^{n}e^{\mu \left(A\right)\left(T_{s}-\sum_{j=1}^{i}{\hat{T}}_{j}\right)}B_{\mu }\left({\hat{T}}_{i},A\right)∥K_{m}∥\\ & \times \prod_{j=1}^{i}\left(e^{\mu \left(A_{m}\right){\hat{T}}_{i-j}}+{B_{m}}_{\mu }\left({\hat{T}}_{i-j},A_{m}\right)∥K_{m}∥\right)e^{\mu \left(A_{m}\right)T_{0}}\\ c_{2}\left(T_{0},{\hat{T}}_{1},...,{\hat{T}}_{n}\right) & =e^{\mu \left(A\right)\left(T_{s}-T_{0}\right)}B_{\mu }\left(T_{0},A\right)+\sum_{i=1}^{n}e^{\mu \left(A\right)\left(T_{s}-\sum_{j=1}^{i}{\hat{T}}_{j}\right)}B_{\mu }\left({\hat{T}}_{i},A\right)∥K_{m}∥\\ & \times \prod_{j=1}^{i}\left(e^{\mu \left(A_{m}\right){\hat{T}}_{i-j}}+{B_{m}}_{\mu }\left({\hat{T}}_{i-j},A_{m}\right)∥K_{m}∥\right){B_{m}}_{\mu }\left(T_{0},A_{m}\right). \end{array}$$

**Proof** **of** **Theorem** **3.** The discretized augmented state $∥\hat{\xi }\left(kT_{s}\right)∥$ can be exponentially bounded such that

$$∥\xi \left(kT_{s}\right)∥\leq \sqrt{\frac{\lambda_{\max}\left(P\right)}{\lambda_{\min}\left(P\right)}}e^{-\hat{\alpha }\left(kT_{s}\right)}∥\xi \left(0\right)∥$$

due to (30). In addition, if it holds that ∥ψ(0)∥=0 (in the initial conditions there is no previous input signal), then

(32) $$∥\xi \left(kT_{s}\right)∥\leq \sqrt{\frac{\lambda_{\max}\left(P\right)}{\lambda_{\min}\left(P\right)}}e^{-\hat{\alpha }\left(kT_{s}\right)}∥\hat{x}\left(0\right)∥.$$

Furthermore, with the same arguments as in Theorem 1, we guarantee that at any time the global exponential stability of the closed-loop system is as follows:

$$\begin{array}{cc} ∥\hat{x}\left(t\right)∥\leq & \left(∥\Pi_{11}^{d}\left(l\tau_{m},{\hat{T}}_{1,l},{\hat{T}}_{2},...,{\hat{T}}_{n}\right)∥+∥\Pi_{21}^{d}\left(l\tau_{m},{\hat{T}}_{1,l},{\hat{T}}_{2},...,{\hat{T}}_{n}\right))∥\right)∥\xi \left(kT_{s}\right)∥\\ & \left(c_{1}\left(l\tau_{m},{\hat{T}}_{1,l},{\hat{T}}_{2},...,{\hat{T}}_{n}\right))+c_{2}\left(l\tau_{m},{\hat{T}}_{1,l},{\hat{T}}_{2},...,{\hat{T}}_{n}\right))\right)∥\xi \left(kT_{s}\right)∥ \end{array}$$

Thus, computing the maximum value of $c_{1}$ and $c_{2}$, (31) is obtained.  ☐

Trivially, Theorem 2 is still valid for the delay case and the optimization can be carried out. Since we now take into account the delay, we perform the optimization in a more realistic way, which helps to maximize the decay rate in a real scenario.

### 3.2. Implementation of Optimization Algorithm in Real-Time Systems

The proliferation of board computers like Raspberry Pi or Beaglebone Black [36,37] together with the emergence of communication networks to connect the elements in a control loop implies that the available computation time might be reduced. For this reason, it is important to limit the computational effort made by the controller in order to guarantee acceptable behavior. In our case, the optimization algorithm can be relaxed in different ways.

A significant source of computations is the discretization of closed-loop system. Since (2) implies solving a matrix integral and since one of the optimization variables is the upper limit of the integral, the optimization algorithm has to execute a hard computation in real time to obtain the discretized model for the corresponding times of application. However, we know the maximum and minimum possible times of application of each signal because they should satisfy (29). Then, we can consider a sufficiently small sampling period *h* in the view of *n* and the sampling period of the plant $T_{s}$ to carry out an offline computation of a discretized matrices grid, i.e., we compute offline $e^{A\left(mh\right)}$ and B(mh,A) for all m∈N such that $0\leq mh\leq T_{s}$. Hence, given the discretized matrices grid, the optimization function should only access the required value instead of computing the corresponding discrete matrix.

The constraints in (23)–(24) might also be a source of delay in the computation. When the times of application $T_{i}$ are taken into account in the optimization, we should consider (23)–(24) as nonlinear constraints of the optimization problem. If there is a large number of input signals, this might constitute a hard computation effort. To avoid this, the optimization algorithm might be solved in two steps. A first step provides the times of application that optimize the decay rate for the auxiliary controller. With fixed times of application, Equations (23)–(24) are transformed in constant limits for the second part of the optimization algorithm, which obtains the actual values of the input signals. Naturally, this results in a suboptimal solution of the problem. In spite of that, the solution always improves the performance of the auxiliary controller.

A final observation in order to minimize the computation time is the consideration of a variable number of input signals to be optimized. Since we know the delay between the sensor and the controller, we can make an estimation of the available computation time and then analyze if it is preferable to reduce the number of input signals (and their corresponding times of application) which are optimization variables, i.e., $n_{\mathrm{opt}}\left(kT_{s}\right)\leq n$, while the rest of the input signals $n-n_{\mathrm{opt}}$ are computed using the auxiliary controller, which typically requires fewer computation resources. We hope to study in detail this possibility in future works in order to increase the algorithm’s applicability.

The implementation of these characteristics in the optimization problem is schematically described in Algorithm 1.

**Algorithm 1** Optimization algorithm with limited computation resources.
**Offline Computation**
Step 1offSynthesis of the auxiliary controller satisfying Assumption 4.Step 2offComputation of the minimum decay rate $\hat{\alpha }$ and the maximum gain $\hat{c}$.Step 3offDiscretization of the closed-loop system for all times mh, m∈N, which satisfy $0\leq mh\leq T_{s}$.

**Online Computation**
Step 1onReception of the output signal $y(kT_{s})$ with a delay $\tau_{sc}\left(kT_{s}\right)$.Step 2onEstimation of the state of the plant from the output.Step 3onDecision of the number of input signals $n_{\mathrm{opt}}$ to be optimized as function of the available computation.Step 4onOptimization of the decay rate as function of the times of application of the input signals maintaining the auxiliary controller.Step 5onComputation of the maximum and minimum bounds to the actual input signals in Assumption 3.Step 6onOptimization of the decay rate as function of the values of the input signals maintaining constant the times obtained in Step 4on.


## 4. Practical Case: Air Levitation System

In this section, we present the experimental results obtained with the optimization algorithm, where we take into account the considerations explained in Section 3.2. We have used the air levitation system proposed in [38] for the experimental validation. Next, a description of the hardware is given. The model of the system and the experimental results are presented afterwards.

### 4.1. Hardware Description

**Figure 2:**
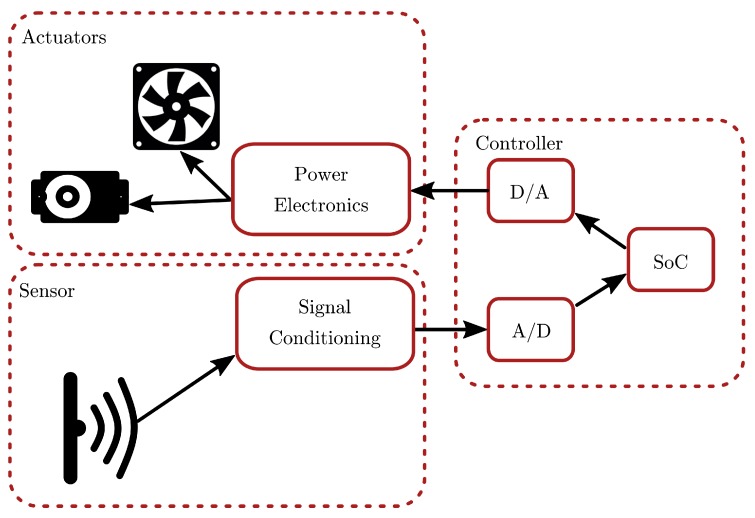
Schematic view of the air levitation control loop.

The air levitation system experimentation platform, based on Arduino Nano, provides two working modes: *standalone*, i.e., the controller is deployed into the Arduino board, and *passthrough*, in which the controller is implemented in the PC and the Arduino board acts mainly as a gateway to interact with the hardware. The system architecture is depicted in Figure 2. The block diagram represents the different functional subsystems: the controller is implemented in the system on a chip (SoC) Arduino board, which also provides digital-to-analog (D/A) and analog-to-digital (A/D) converters. Additional circuitry is required to condition the sensor readings, and to amplify the control signal in order to provide enough energy for the actuators (fan and servo).

To measure the position of the ball, several possibilities were considered: visual recognition, ultrasonic sensors, and infrared sensors. While it could be interesting to use a video cam to get the ball position, and it may even be adequate for teaching with an image-processing subject, the complexity and cost of the system would increase, so this was discarded as a possibility. With respect to the ultrasonic distance sensors, they are a valid alternative to infrared ones, and are similar in cost and complexity. However, the latter option was finally chosen. The position of the ball is measured with an infrared beam sensor, specifically a Sharp GP2Y0A21YK0F Analog Distance Sensor (Sharp Corporation), which can obtain measurements between 10 and 80 cm. There are other similar models that are electrically compatible and have different ranges, such as the GP2Y0A21YK0F (4–30 cm) and the GP2Y0A02YK0F (20–150 cm), and thus a choice can be made in order to adapt to different tube lengths. All the aforementioned sensors are analog, yielding a signal roughly in the range of 0–5 V, which is proportional to the inverse of the distance measured. The sensor is composed of two infrared LEDs, an emitter which projects a light beam, and a receiver that measures the bounce in the detected object. Since the sensor measures the light reflected by the object, it may be affected by the color, shape and movement of the object. Also, it has an update period of approximately 40 ms. These aspects must be taken into account to get reliable measures.

The fan speed control, as well as the servo position, are achieved through *Pulse Width Modulation* (PWM). The fan speed, and consequently the air flow, can be controlled with a high frequency PWM. Also, most digital interfaced servomotors admit a PWM signal, with a carrier frequency of 20 HZ, which codes the angular position into the duty cycle, though it is not the same for all models. For example, many servos provide a mapping between (1 ms, 2 ms)↦(0${}^{\circ }$, 180${}^{\circ }$). While Arduino provides built-in PWM outputs, these provide limited current, so there is a need for additional components, as mentioned before.

The *hardware interface* subsystem is in charge of reading measures from the sensors and sending values to the actuators. Though it is obviously platform-dependent, it is good practice to use standard libraries and protocols. For example, the Arduino application programming interface (API) is widely used for its simplicity and it has been exported to other hardware, like the Beaglebone boards or Raspberry PI. The functionality to be covered can usually be reduced to read and write digital or analog input and outputs. In the air levitation system, the hardware interface task is accomplished using *Javascript Object Notation* (JSON), which is human-friendly (it can be easily read), and at the same time can be processed by most programming languages.

There is a *real-time loop* implementing the time-critical actions: reading sensors, updating the controller, and writing outputs. A lower priority loop is responsible for two tasks: sending measurements and logs to the PC and receiving user commands (for example, to change the setpoint or other parameters). Once the values have been acquired, it is important to store them in order to be accessed whenever is required. The *datalogging* capabilities have been separated into a low priority task that periodically dumps measures and control actions to a database, so the data is stored and can be accessed to perform off-line processing of past sessions.

The *control* subsystem implements a *proportional–integral–derivative PID* controller for which parameters can be modified and tuned. The control subsystem is prepared to be extended with more sophisticated controllers without much development effort. Apart from this PID implementation and template, a passthrough mode is provided that can be used to use the user PC as a controller. This is convenient for research, for example to test a prototype developed in MATLAB, or when there are high computation power needs.

### 4.2. System Model

**Figure 3:**
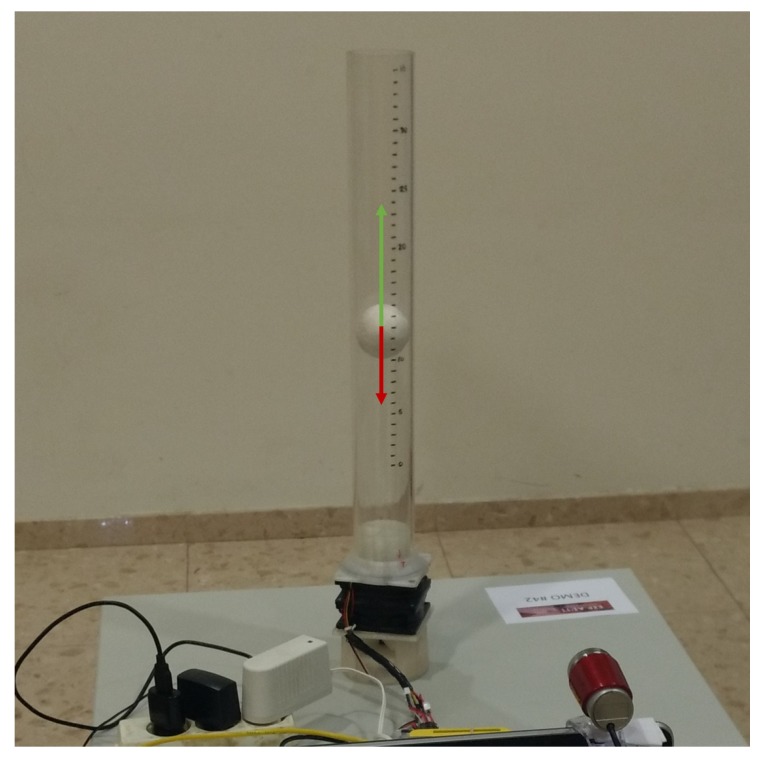
Balance of forces of the air levitation system used in the experiment. The red arrow shows the gravitational force, while the green arrow shows the force applied by the air flow.

The only forces acting over the levitating object are the upward effect of the air flow, and the downwards effect of the gravity, as shown in Figure 3. According to this, Newton’s second law gives the nonlinear model

(33) $$m\Delta \ddot{z}\left(t\right)=\frac{1}{2}C_{d}\rho A{\left(v_{w}-\dot{z}\left(t\right)\right)}^{2}-mg,$$

where *m* is the mass of the object to levitate, *z* is the vertical position of the object in the tube, ρ is the density of air, *A* is the object’s area exposed to the upwards air flow, $v_{w}$ is the velocity of the air inside the tube, *g* is the gravitational acceleration, and $C_{d}$ is the drag coefficient.

Following [38], a linearized system from (33) can be obtained with matrices

$$\begin{array}{cc} A_{p}=\left(\begin{array}{ccc} -8.52 & -2.97 & 0\\ 4.00 & 0 & 0\\ 0 & 1.00 & 0 \end{array}\right),\;B_{p}=\left(\begin{array}{c} 2.00\\ 0\\ 0 \end{array}\right), & C_{p}=\left(\begin{array}{ccc} 0 & 0 & 3.36 \end{array}\right),\;D_{p}=0. \end{array}$$

We use this model to solve the optimization problem. The consideration of the times of application as variables of the optimization problem provides not only more degrees of freedom in the solutions but also a better treatment of the delays, since $T_{1}$ and hence the first imput signal $u_{1}$, can be computed accordingly. As auxiliary controller, we make use of a PI controller with gains $K_{p}=0.006$ and $K_{c}=0.002$. We consider a sampling period of the output $T_{s}=300$ ms. We assume that there is a constant computation delay $\tau_{\mathrm{com}}=50$ ms and there is a time-varying delay because of the communication through the network, which is bounded by $\tau_{M,\mathrm{net}}=25$ ms. We consider n=2, i.e., the input signal is changed twice in each $T_{s}$. Applying Theorem 3, we obtain $\hat{\alpha }=0.0017$ and $\hat{c}=734.24$.

### 4.3. Experimental Results

**Figure 4:**
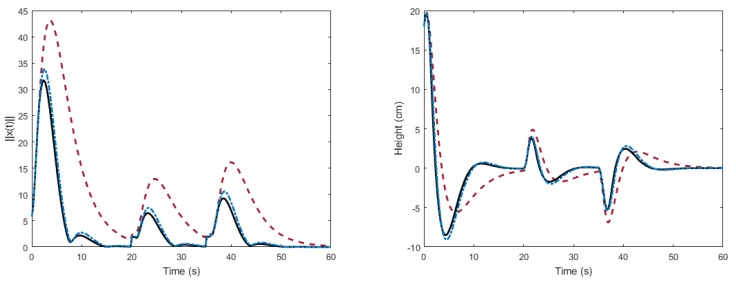
Norm of the state vector (**left**) and output response (**right**) of the linear model of the air levitation system. Solid black line: the proposed algorithm. Dot-dashed blue line: the algorithm in [30]. Dashed red line: auxiliary PI controller.

**Figure 5:**
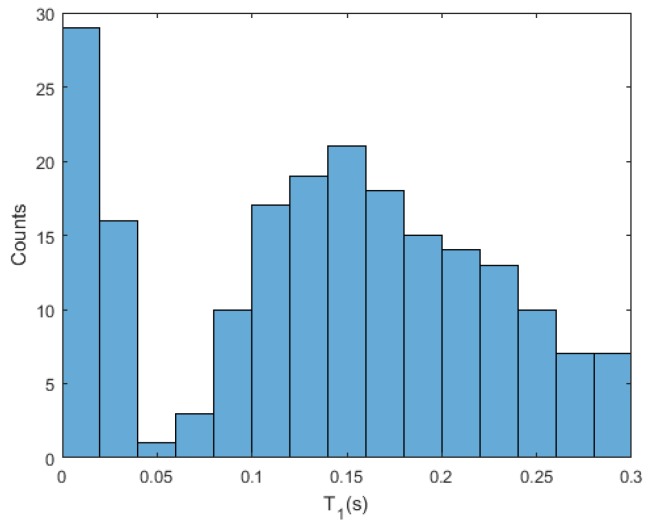
Histogram of times of application of $u_{1}$ in the linear model of the air levitation system.

Firstly, as a preliminary test of the validity of the developed theory, we carry out a simulation of the linear model. In the simulation, we stabilize the state of the system in the origin. As shown in Figure 4, the decay rate is considerably improved using the optimization algorithm with respect to the auxiliary PI controller. In addition, we can further improve the results by performing the optimization as function of the times of application. The novel algorithm allows the use of the knowledge of the delays to appropriately choose the times of application. This can be observed in the histogram of Figure 5.

**Figure 6:**
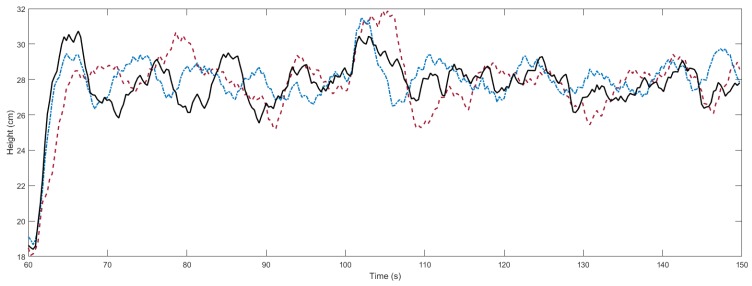
Output response of the air levitation system in the stabilization experiment. Solid black line: proposed algorithm. Dot-dashed blue line: algorithm in [30]. Dashed red line: auxiliary PI controller.

**Figure 7:**
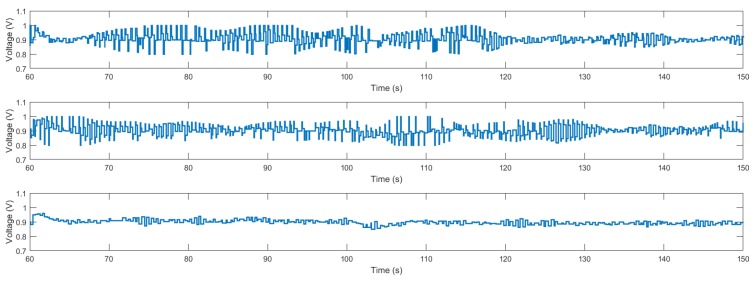
Input voltage applied to the air levitation system in the stabilization experiment. From top to bottom: the proposed algorithm, the algorithm in [30], and the auxiliary PI controller.

**Figure 8:**
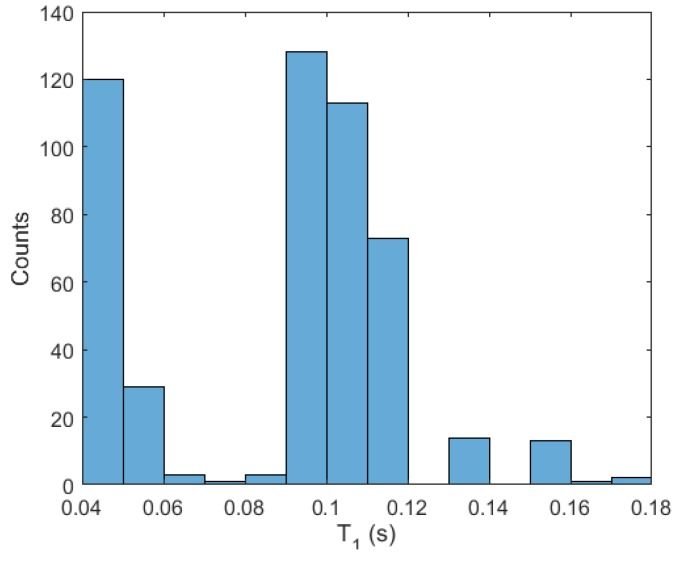
Histogram of times of application of $u_{1}$ in the real system.

Secondly, we perform two experiments in the real system. In Figure 6, we can observe the results obtained with the proposed algorithm with respect to the auxiliary PI controller and with respect to the optimization algorithm considering constant times of application. In the experiment, the signal reference is set to 18 cm, and later two reference changes are made. At 60 s the reference is set to 28 cm, and at 100 s a disturbance is introduced into the servo. The input signals are shown in Figure 7. The optimization of the decay rate implies a more aggressive input. In spite of the inherent limitations (sensor sensitivity, disturbances, model errors, etc.) of the experimental system, which contribute to complicate the optimization, the proposed algorithm provides improvements during the stabilization in measure of the integral square error (ISE) of 31.77% and 5.35%, and in the settling time of 42.86% and 20.00% with respect to the auxiliary controller and the algorithm in [30], respectively, as shown in Table 1. In the histogram of Figure 8, we observe the distribution of times of application of input signal $u_{1}$. The times of application of $u_{2}$ can be computed taking into account the delay.

**Figure 9:**
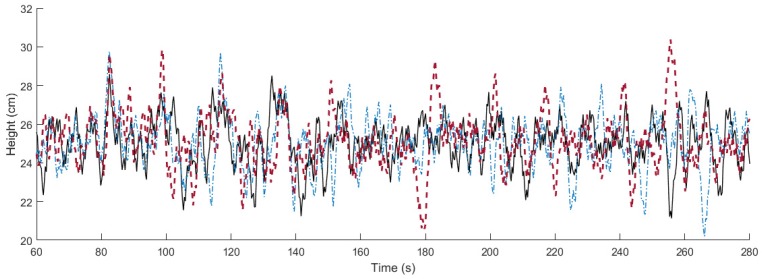
Output response of the air levitation system in the disturbance rejection experiment. Solid black line: proposed algorithm. Dot-dashed blue line: algorithm in [30]. Dashed red line: auxiliary PI controller.

**Figure 10:**
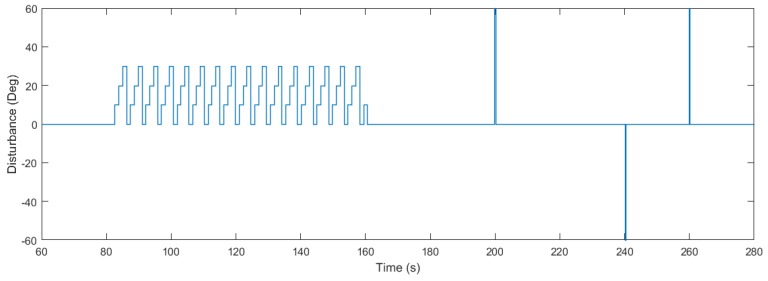
Disturbance applied to the system. Solid black line: proposed algorithm. Dot-dashed blue line: algorithm in [30]. Dashed red line: auxiliary PI controller.

**Figure 11:**
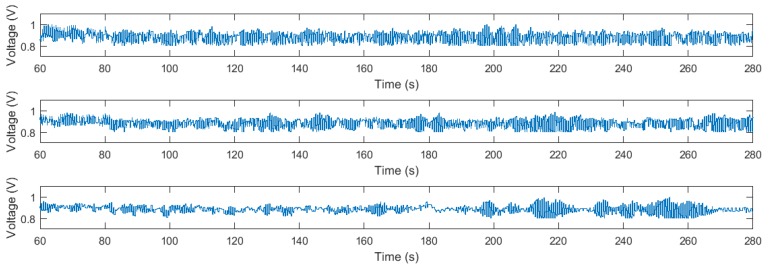
Input voltage applied to the air levitation system in the disturbance rejection experiment. From top to bottom: the proposed algorithm, the algorithm in [30], and the auxiliary PI controller.

In Figure 9, the response of the system is represented for the three different methods when different disturbances are applied. Specifically, we apply a disturbance with form of sawtooth wave between 80 and 160 s. Later, we apply several impulse disturbances between 200 and 260 s, as shown in Figure 10. Similarly to the first experiment, the optimization methods cause more changes in the input signal (see Figure 11), which is the price to pay for maximizing the decay rate. Table 1 gathers the information about the ISE during the different disturbances, obtaining improvements of 27.65% and 13.59% in the sawtooth wave disturbance rejection with respect to the auxiliary controller and the algorithm in [30], respectively, and of 33.01% and 27.37% in the impulse disturbance rejection, respectively.

## 5. Discussion

In this paper, we propose an optimization algorithm for a multi-rate input control system. The algorithm is designed to be implemented in real networked control systems. For this reason, the algorithm is designed to take into account time-varying delays. The proposed method presents some advantages with respect to to the existing literature. It takes into account the times of application and the time-varying delays in the optimization problem as opposed to [19,20,21,22,23,24]. In addition, these variable times of application cause irregular sampling, as considered in [3,4,5], but with the difference that the irregular sampling in the proposed method is motivated by a theoretical criterion to maximize the decay rate, and in [3,4,5] it is just a tuning parameter. In contrast, the computing time of the algorithm might be considerably large depending on the number of control signals and time of application to be optimized. Some solutions, such as precomputing the discretization of the matrices, are provided to reduce the computational load of the algorithm in order to be implemented in single-board computers. These solutions enable the implementation of the method in a larger number of plants. As an example, we successfully apply the control scheme to an air levitation system, while the algorithm implemented without these consideration, such as in [30], requires a larger computing time and the control objective cannot be achieved. Despite this, the proposed algorithm always induces a larger delay than the associated auxiliary controller. Hence, its implementation in fast systems should be carefully considered. Both the theoretical and experimental results show that the proposed algorithm allows a faster response of the system while dealing with network delays.

The algorithm could be improved in several ways. A more accurate estimation of the delays, or even some kind of disturbance estimation, would provide a better model for the optimization and, naturally, an improvement in the system response. As aforementioned, the main issue of the algorithm is the delay that it induces in the system due to the running time. For this reason, novel methods to reduce the running time might be a way to improve the results of the algorithm. Finally, in this paper we consider a dual-rate scheme with fast input, but future works might include other multi-rate configurations.

## Figures and Tables

**Table 1:** Comparison of different controllers.

Controller	Settling Time	ISE (Stabilization)	ISE (Sawtooth Wave)	ISE (Impulse)
Auxiliary PI controller	4.2 s	421.39	291.01	172.43
Algorithm in [30]	3.0 s	303.78	243.64	159.03
Proposed algorithm	2.4 s	287.53	210.54	115.51

## Appendix A

First of all, we observe that if the estimation model coincides with the actual plant, (15) can be written as

$$\Pi =\prod_{i=1}^{n}e^{A{\hat{T}}_{i}}+B\left({\hat{T}}_{i},A\right)K.$$

Consequently,

$$\begin{array}{cc} c({\hat{T}}_{1},...,{\hat{T}}_{n}) & =\sqrt{\frac{\lambda_{\max}\left(P\right)}{\lambda_{\min}\left(P\right)}}e^{\hat{\alpha }T_{s}}\prod_{i=1}^{n}\left(e^{\mu \left(A\right){\hat{T}}_{i}}\right.+B_{\mu }\left({\hat{T}}_{i},A\right)∥K∥). \end{array}$$

For simplicity, let us consider the two first control actions. They are applied to the plant during periods ${\hat{T}}_{1}$ and ${\hat{T}}_{2}$, respectively, such that ${\hat{T}}_{1}+{\hat{T}}_{2}={\hat{T}}_{12}$. We can search the maximum of

$$c_{12}\left(\hat{T_{1}}\right)=\left(e^{\mu \left(A\right)\left({\hat{T}}_{12}-{\hat{T}}_{1}\right)}+B_{\mu }\left(\left({\hat{T}}_{12}-{\hat{T}}_{1}\right),A\right)∥K∥\right)\left(e^{\mu \left(A\right){\hat{T}}_{1}}+B_{\mu }\left({\hat{T}}_{1},A\right)∥K∥\right),$$

which can be considered independently of the rest of the actuation periods. The derivative of $c_{12}$ results in

$$\begin{array}{cc} \frac{dc_{12}\left({\hat{T}}_{1}\right)}{d{\hat{T}}_{1}} & =\left(\mu (A)+∥B∥∥K∥\right)e^{\mu \left(A\right)\left({\hat{T}}_{12}-{\hat{T}}_{1}\right)}\left(e^{\mu \left(A\right){\hat{T}}_{1}}+B_{\mu }\left({\hat{T}}_{1},A\right)∥K∥\right)\\ +\left(e^{\mu \left(A\right)\left({\hat{T}}_{12}-{\hat{T}}_{1}\right)}+B_{\mu }\left(\left({\hat{T}}_{12}-{\hat{T}}_{1}\right),A\right)∥K∥\right)\left(\mu (A)+∥B∥∥K∥\right)e^{\mu \left(A\right)\left({\hat{T}}_{1}\right)}, \end{array}$$

which has an unique critical point (the maximum) in ${\hat{T}}_{1}={\hat{T}}_{12}/2$ and, logically, ${\hat{T}}_{2}={\hat{T}}_{1}$. If we now consider the period ${\hat{T}}_{1}+{\hat{T}}_{2}+{\hat{T}}_{3}={\hat{T}}_{13}$, we can use the above argument to write ${\hat{T}}_{1}=\left({\hat{T}}_{13}-{\hat{T}}_{3}\right)/2$ and ${\hat{T}}_{3}=\left({\hat{T}}_{13}-{\hat{T}}_{1}\right)/2$. Hence, ${\hat{T}}_{1}={\hat{T}}_{2}={\hat{T}}_{3}={\hat{T}}_{13}/3$. Finally, we can use the argument recursively to prove that $c\left({\hat{T}}_{1},...,{\hat{T}}_{n}\right)\leq c\left(T_{s}/n,...,T_{s}/n\right)$. □
